# Functional variant rs12614 in *CFB* confers a low risk of IgA nephropathy by attenuating complement alternative pathway activation in Han Chinese

**DOI:** 10.3389/fimmu.2022.973169

**Published:** 2022-10-13

**Authors:** Dian-Chun Shi, Shao-Zhen Feng, Zhong Zhong, Lu Cai, Meng Wang, Dong-Ying Fu, Xue-Qing Yu, Ming Li

**Affiliations:** ^1^ Department of Nephrology, The First Affiliated Hospital of Sun Yat-sen University, Guangzhou, China; ^2^ NHC Key Laboratory of Clinical Nephrology (Sun Yat-sen University) and Guangdong Provincial Key Laboratory of Nephrology, Guangzhou, China; ^3^ Division of Nephrology, Guangdong Provincial People’s Hospital, Guangdong Academy of Medical Sciences, Guangzhou, China

**Keywords:** IgA nephropathy, complement factor B, case-control study, alternative pathway, polymorphism

## Abstract

Activation of the alternative pathway (AP) of complement is thought to play an important role in Immunoglobin A nephropathy (IgAN). Our previous study showed that rs4151657 within the complement factor B (*CFB*) gene increased the risk of IgAN. The protein encoded by the *CFB* gene is an initial factor that promotes AP activation. The aim of this study was to investigate whether other variants of *CFB* confer susceptibility to IgAN and elucidate their potential roles in AP activation. A total of 1,350 patients with IgAN and 1,420 healthy controls were enrolled and five tag single-nucleotide polymorphisms were selected for genotyping. The levels of key AP components, such as CFB, complement factor H and complement split product C3a, were measured by enzyme-linked immunosorbent assay. Molecular docking and molecular dynamic simulation were carried out to characterize the mutation of residues in the protein structure and the dynamic properties of wide type and mutation models of CFB protein. The allele-specific effect on CFB expression and its binding affinity to C3b were investigated through cell transfection and surface plasmon resonance analysis, respectively. We found that rs12614 significantly reduced the risk of IgAN (OR = 0.69, 95% CI = 0.52–0.91, *P* = 0.009), and the rs12614-T (R32W mutation) was correlated with lower CFB levels, higher serum C3 level, and less mesangial C3 deposition in patients with IgAN. The structural model showed that the R32W mutation reduced the structural stability of CFB protein. Furthermore, *in vitro* study revealed that rs12614-T decreased the expression of CFB and reduced its binding affinity to C3b by four-fold compared with rs12614-C. In conclusion, the rs12614-T in *CFB* was associated with low risk of IgAN probably by attenuating AP activation.

## Introduction

Immunoglobin A nephropathy (IgAN) is one of the most common prevalent primary glomerulonephritis throughout the world. It is characterized by the mesangial deposition of immune complexes, which are predominantly composed of IgA1 and complement C3 proteins (1, 2). IgAN is the major cause of end-stage renal disease in China, and approximately 20%–50% of patients will require renal replacement therapy within 20 years after their initial diagnosis (3, 4). Although the pathogenesis of IgAN remains unclear, the alternative pathway (AP) of complement was considered to play an essential role of IgAN (5–7). Some key components of the AP, including complement factor B (CFB), complement factor H (CFH), complement factor P and Ba, are not only increased in peripheral blood but also codeposited in the mesangium of patients with IgAN, indicating that dysregulated complement AP activation contributes to the development of IgAN (8–10).

Some evidence from genetic perspective also supports the importance of complement AP activation in IgAN. Two genome-wide association studies (GWASs) of IgAN performed in a European population revealed 1q32 containing the *CFH* gene as the susceptibility locus for IgAN (11, 12). Recently, our case-control study conducted in a Chinese Han population found that single-nucleotide polymorphism (SNP) rs4151657 in the *CFB* gene significantly increased the risk of IgAN and might affect complement AP activation in patients with IgAN (13). The *CFB* gene was also demonstrated to potentially confer susceptibility to IgAN in an exome array study (14). CFB is a glycoprotein encoded by the *CFB* gene and is considered as the main initiation factor of AP activation. As a vital positive molecule, CFB could integrate with complement protein C3b to form C3 convertase, which is a key step in complement AP activation (15, 16). In view of the importance of CFB protein in IgAN, the use of an oral CFB inhibitor named LNP023 is ongoing in a phase III clinical trials (NCT04578834) in patients with IgAN, and this is expected to be the first AP inhibitor applied in the treatment of IgAN (17). In addition, other functional variants of L433S, F286L, and K323E within *CFB* were reported to be involved the development of atypical hemolytic uremic syndrome (aHUS) by affecting the formation of C3 convertase and activation of complement AP (18, 19).

Accumulating evidence suggests that genetic variants of *CFB* may be involved in the development and progression of IgAN. However, there are no comprehensive study about the relationships between variants of *CFB* and the risk of IgAN until now. Therefore, we performed this case-control association study to explore whether there were additional variants of the *CFB* gene conferred the risk of IgAN and further elucidated their potential roles in the mechanism of IgAN in a Chinese Han population.

## Materials and methods

### Study subjects

A total of 1,350 sporadic patients with biopsy‐proven IgAN were recruited from the Department of Nephrology, The First Affiliated Hospital of Sun Yat-sen University. Patients with Henoch-Schonlein purpura, systemic lupus erythematosus, HIV infection, diabetes, and hepatitis B-associated glomerulonephritis were excluded. The 1,420 age- and gender-matched unrelated individuals, who were verified with no history of renal disease and normal urinalysis, were enrolled as healthy controls. All patients with IgAN and healthy controls were self-reported Han Chinese. The baseline demographic and clinical information of patients with IgAN were collected at the time of diagnosis. This case-control study was conducted in compliance with the Helsinki Declaration and was approved by the Ethics Committee of The First Affiliated Hospital, Sun Yat-sen University (No. 2016-215). All subjects provided written consents.

### SNP selection and genotyping

The location of the *CFB* gene was mapped to chromosome 6, position 31913721-31919861 (hg19, data obtained from the UCSC, February 2009 assembly). SNP genotyping information of this gene was obtained from the genotyped SNP database of the Chinese Han Beijing population in the 1000 Genomes Project (http://www.1000genomes.org). Haploview 4.2 software was used to analyze the genotype data retrieved from the 1000 Genomes Project, and five tag SNPs (rs1048709, rs12614, rs2072633, rs537160, and rs541862) in the *CFB* gene were selected with the following criteria: minor allele frequency ≥ 5% and pairwise linkage disequilibrium of *r^2^* threshold of 0.80. Finally, a total of five tag SNPs were chosen for further association analysis (**Supplementary Table 1**).

The genomic DNA was isolated from the whole blood of all the subjects by using a DNA extraction kit (Qiagen, Hilden, Germany) in accordance with the manufacturer’s protocol. Kompetitive Alelle Specific PCR technology with SNPLine platform (applied by Shanghai Baygene Biotechnology Company Limited, Shanghai, China) was used for genotyping. The specific primers labeled with 5-carboxyfluorescein or 5-hexachloro-fluorescein were designed for each variant (LGC Genomics, UK) and were listed in **Supplementary Table 2**.

### Measurement of the serum levels of key AP components

Patients with primary IgAN who were treated with glucocorticoids or other immunosuppressant treatment within the past 6 months or had a history of infection within the past 30 days before enrollment were excluded. The serum concentrations of CFB, CFH, and C3a from 71 patients with IgAN and 59 healthy controls were individually detected by using a commercial enzyme-linked immunosorbent assay (ELISA) kit (USCN Life Science, Wuhan, China).

### SNP function prediction

The RegulomeDB web database (http://www.regulomedb.org/) was used to calculate the functionality score for R32W. In addition, PolyPhen-2 (http://genetics.bwh.harvard.edu/) and SIFT (http://sift.jcvi.org/) were also used to investigate the potential effect of missense mutation on protein function.

### Structure modeling and molecular docking

The X-ray structures of wild-type (WT) CFB were downloaded from RCSB Protein Data Bank (PDB ID: 2OK5). The protein structure of R32W mutation was constructed in Molecular Operating Environment (MOE) software (version 2018.01) (20). We constructed the mutated structure and performed subsequent energy minimization based on the structure of WT CFB. The energy minimization was carried out with the Amber12: EHT force field, which allows atoms in the mutated residue and neighboring residues within 10 Å to be free to move and maintains other atoms in a fixed position. The structures of the above two proteins (WT and R32W) were optimized by molecular dynamics simulation using AMBER16 software (21). Each protein structure was neutralized by adding sodium/chlorine counter ions and solvated in a cuboid box of TIP3P water molecules with solvent layers 10 Å between the box edges and solute surface. Protein–protein docking in ClusPro server was used for molecular docking simulations to determine the ability of WT CFB and mutant CFB to interact with C3b.

### Transient transfection and CFB expression assays

A eukaryotic expression plasmid containing the coding sequence of *CFB*, either the rs12614-C allele (CS-Q0537-M35) or the rs12614-T allele (EX-Q0537-M35), was constructed (GeneCopoeia, MD, USA). The expressed proteins were WT CFB_32R_ and mutant CFB_32W_, respectively. Human embryonic kidney 293 (HEK293, ATCC, Manassas, VA, USA) cells were maintained in antibiotic-free Dulbecco’s modified Eagle’s medium (Gibco) supplemented with 10% fetal bovine serum (Gibco). The cells were seeded into 12-well plates and transfected with 500 ng of each plasmid DNA by using a Lipofectamine 3000 kit (Life Technologies, Carlsbad, CA, USA) according to the manufacturer’s instructions. The empty vector pReceiver-M35 was taken as a control. After 48 h of transfection, the cell supernatants were harvested after centrifugation. The CFB levels in the cell supernatants were analyzed by an ELISA kit (Abcam, Cambridge, UK). Each test included two biological replicates for each group, and the experiment was repeated three times.

### Surface plasmon resonance analysis of C3 convertase formation

The recombinant WT (32R) and mutant CFB (R32W) were overexpressed and purified from HEK293 cells by the Novoprotein Scientific Inc. (Shanghai, China). The interaction of CFB with C3b was analyzed by using surface plasmon resonance (SPR) technology with Biacore T1000 equipment (GE Healthcare). C3b (Millipore, MA, USA) was coupled to the sensor CM5 (carboxymethylated dextran) chip according to the standard amide-coupling technology. To analyze the effect of mutation on proenzyme formation of C3 convertase (C3bB), the WT and mutant CFB were individually flowed across the C3b-bearing surface at various concentrations (0.125, 0.25, 0.5, 1.0, 2.0, and 4.0 μM) and injected at 30 μl/min in a HEPES (4-(2-hydroxyerhyl) piperazine-1-erhanesulfonic acid) buffer [10 mM HEPES (pH 7.4), 50 mM NaCl, 3 mM MgCl_2_, and 0.005% surfactant P20]. To further analyze the effect of mutation on formation of activated C3 convertase (C3bBb), recombinant CFB was used as an analyte at 1.0, 1.5, 2.0, 3.0, and 4.0 μM in HEPES buffer with 1 mM complement factor D (CFD) [10 mM HEPES (pH 7.4), 50 mM NaCl, 3 mM MgCl_2_, 1 mM CFD, and 0.005% surfactant P20]. An empty flow cell with HEPES buffer was used as a control. The data were assessed by Biacore T100 evaluation software (version 1.1), and the resonance units from the blank flow cell were subtracted. Kinetic parameters fitted to all curves were determined by fitting the obtained sensorgrams into two state interaction models as previously described (22).

### Statistical analyses

The allele and genotype frequencies of *CFB* polymorphisms were obtained by direct counts. The Hardy–Weinberg equilibrium (HWE) was assessed among the five SNPs using the chi-square test, and SNPs consistent with the HWE in controls were included for further analysis. Three genetic models (additive, dominant, and recessive) were used to explore the genetic associations between tag SNPs and IgAN risk, and these associations were evaluated by odds ratios (ORs) and 95% confidence intervals (CIs) after adjusting for age and gender as previously described (23). The major allele was considered as the reference allele. For individual SNP analysis, Bonferroni correction was used for multiple adjustments, and *P* < 0.01 indicated statistical significance. We used Haploview 4.2 software (Broad Institute, Cambridge, MA, USA) to investigate the linkage disequilibrium patterns of candidate SNPs of *CFB* and to analyze the association between haplotypes and IgAN risk by using 1,000 permutation tests (24). PLINK software (version 1.09) was used to evaluate the genetic associations of *CFB* polymorphisms with IgAN susceptibility.

The mean ± standard deviation was used to describe data with the normal distributions, and the median (interquartile range) and percentages were used to present data with skewed distributions and categorical variables, respectively. Student’s *t*-test and non-parametric Mann-Whitney *U*-test were conducted for comparison of continuous variables, and the chi-square test was performed for comparison of categorical variables. The Cox proportional hazard model was used for survival analysis. Statistical analysis was conducted using SPSS Statistics, version 21 (SPSS, Chicago, IL, USA) and GraphPad Prism 6.0 software (GraphPad Software Inc., San Diego, CA, USA).

## Results

### Basic characteristics of the participants

We recruited 1,350 patients with IgAN and 1,420 normal controls in this study. A total of 1,333 patients with IgAN and 1,413 normal controls were included for further analysis after quality control, and 24 individuals (17 patients and seven controls) were excluded because of poor genotyping performance. The general information of all the participants is shown in **Supplementary Table 3**. No significant differences in sex (*P* = 0.598) or age (*P* = 0.486) were observed between patients with IgAN and normal controls.

### Associations between *CFB* gene polymorphisms and IgAN susceptibility

All tag SNPs were successfully genotyped with the call rates > 95%, and none of the SNPs violated HWE in the controls (*P* > 0.05). The allele and genotype frequencies of five tag SNPs in patients with IgAN and normal controls are shown in **Table 1**. Among the five SNPs, only rs12614 was found to be significantly associated with the susceptibility of IgAN under the dominant model (rs12614-TT+TC versus rs12614-CC) after Bonferroni’s correction (OR = 0.69, 95% CI = 0.52–0.91, *P* = 0.009) (**Table 1**).

**Table 1:** Associations of *CFB* gene polymorphisms with the risk of IgAN.

SNP	Allele(A/B)^a^	Genotype (AA/AB/BB)			*P*_HWE_	Additive		Dominant		Recessive	
		Cases (N = 1,333)	Controls (N = 1,413)	MAF^b^		OR (95%CI)	*P*	OR (95%CI)	*P*	OR (95%CI)	*P*
rs1048709	A/G	119/554/660	118/585/710	0.30/0.29	0.87	1.03 (0.92–1.16)	0.603	1.03 (0.89–1.19)	0.716	1.08 (0.83–1.41)	0.587
rs12614	T/C	4/87/1242	3/132/1278	0.04/0.05	0.83	0.72 (0.55–1.00)	0.016	0.69 (0.52–0.91)	**0.009**	1.39 (0.31–6.23)	0.665
rs2072633	C/T	268/681/384	313/716/384	0.46/0.47	0.55	0.93 (0.83–1.03)	0.170	0.92 (0.78–1.09)	0.344	0.89 (0.74–1.06)	0.193
rs537160	T/C	262/670/401	276/694/443	0.45/0.44	0.89	1.03 (0.93–1.15)	0.601	1.06 (0.90–1.25)	0.476	1.01 (0.84–1.22)	0.924
rs541862	G/A	3/139/1191	3/147/1263	0.05/0.05	0.55	1.00 (0.79–1.27)	0.983	1.00 (0.79–1.28)	0.992	1.07 (0.22–5.33)	0.932

Bold characters indicated reaching Bonferroni significant threshold for associations (P = 0.01).

a A: minor allele/B: major allele.

b Minor allele frequency (MAF) among cases/controls.

IgAN, IgA nephropathy; SNP, single-nucleotide polymorphism; MAF, minor allele frequency; HWE, Hardy–Weinberg equilibrium; OR, odds ratio; CI, confidence interval.

Furthermore, the relationship of *CFB* haplotypes with the risk of IgAN was also evaluated, and the linkage disequilibrium block on *CFB* was constructed by rs537160, rs541862, rs4151657, and rs2072633 in chromosome 6 (**Supplementary Figure 1**). The haplotype analysis showed that the haplotype “CATC” was correlated with a decreased risk of IgAN (OR = 0.75, 95% CI = 0.64–0.87, *P* = 0.002) (**Supplementary Table 4**). However, the SNPs included in the CATC haplotype were not in highly linkage with the rs12614, in which the r^2^ of rs12614 with rs537160, rs541862, rs4151657, and rs2072633 were 0.03, 0, 0.01, and 0.04, respectively (see details in **Supplementary Figure 1**).

### Associations of rs12614 with clinicopathological phenotypes in IgAN

We further investigated the correlation of rs12614 with the clinicopathological phenotypes and prognosis of IgAN. The results suggested that the individuals carrying the protective allele T of rs12614 had increased circulating C3 levels (*P* = 0.011) and less intense mesangial C3 deposition (*P* = 0.019) (**Table 2**), in which both were the indicators of milder complement activation.

**Table 2:** Associations of SNP rs12614 with clinicopathological parameters in IgAN cases.

Parameters	CC (N = 1,242)	TC+TT (N = 91)	*P*
Hypertension (n, %)	440 (35.42%)	31 (34.07%)	0.793
Serum creatinine (μmol/L)	108.00 (73.00, 204.50)	100.00 (69.00, 185.00)	0.305
eGFR(ml/min/1.73m^2^)	64.60 (30.22, 96.24)	71.61 (33.37, 100.21)	0.479
Serum C3^a^ level (g/L) (n, %)			**0.011**
≥0.79 g/L	921 (76.88%)	78 (88.64%)	
<0.79 g/L	277 (23.12%)	10 (11.36%)	
Serum IgA level^b^ (g/L) (n, %)			
>3.45 g/L	343 (28.68%)	24 (27.27%)	0.778
≤3.45 g/L	853 (71.32%)	64 (72.73%)	
Urine protein (g/day)	1.35 (0.55–2.92)	1.44 (0.73–3.21)	0.358
Hyperuricemia	681 (56.05%)	49 (53.85%)	0.683
Hyperlipemia	900 (74.68%)	68 (76.40%)	0.683
Mesangial C3 deposition			**0.019**
0	247 (19.92%)	27 (29.67%)	
±~1+	365 (29.44%)	27 (29.67%)	
2+	526 (42.42%)	33 (36.26%)	
3+~4+	102 (8.23%)	4 (4.40%)	
Mesangial hypercellularity (M1) (n, %)	681 (54.83%)	52 (57.14%)	0.669
Endocapillary hypercellularity (E1) (n, %)	216 (17.39%)	19 (20.88%)	0.399
Segmental glomerulosclerosis (S1) (n, %)	678 (54.59%)	47 (51.65%)	0.587
Tubular atrophy/Interstitial fibrosis (n, %)			0.874
T0	726(58.45%)	54 (59.34%)	
T1	321 (25.85%)	23 (25.27%)	
T2	195 (15.70%)	14 (15.38%)	

SD, standard deviation; eGFR, estimated glomerular filtration rate.

Bold characters indicated significant associations (P < 0.05).

a 0.79 g/L is the lower limit of serum C3 level according to our hospital reference range.

b 3.45 g/L is the higher limit of serum IgA level according to our hospital reference range.

Among 1,333 patients with IgAN, longitudinal retrospective progression data were available for 660 individuals with a median follow-up time of 26.7 months. In total, 91 patients with IgAN reached the endpoint, which was considered as end-stage renal disease or doubling of serum creatinine levels. Cox regression analysis showed no significant association between rs12614 and renal survival of patients with IgAN (**Supplementary Table 5**).

### Comparisons of the serum levels of key AP components with different genotypes of rs12614

**Figure 1:**
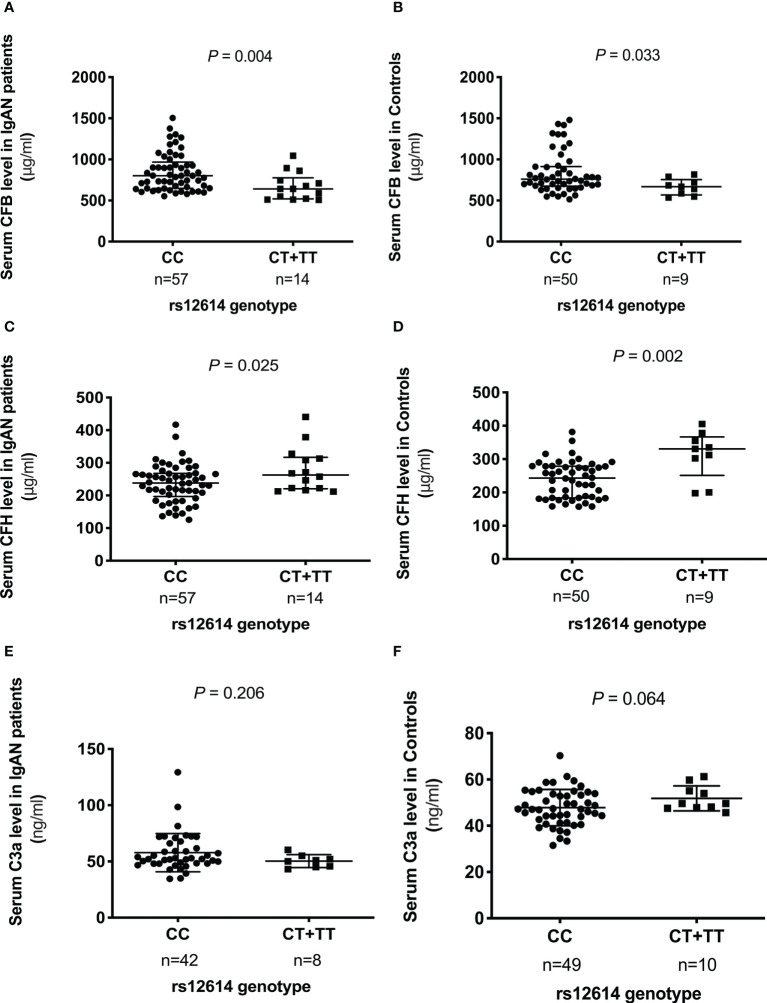
Comparison of serum levels of CFB, CFH, and C3a among different genotypes of rs12614. The serum levels of CFB **(A, B)**, CFH **(C, D)**, and C3a **(E, F)** in patients with IgAN and healthy controls among different genotypes of rs12614 (CC or CT+TT). The bars represent the mean (standard deviation) or median (interquartile range). The *P*-value was determined by Student’s *t*-test or the non-parametric Mann-Whitney *U*-test.

To evaluate the effect of rs12614 on the expression of the key AP components, the serum levels of CFB, CFH, and C3a were measured (**Figure 1**). Either patients with IgAN or normal controls carrying the protective allele T (TT or CT genotypes) had significant lower CFB levels than those harboring the CC genotype [TT+CT versus CC: 640.7 (519.7–775.2) μg/ml versus 800.1 (648.1–966.9) μg/ml in patients with IgAN, *P* = 0.004; 668.0 (566.7–754.3) μg/ml versus 759.2 (673.6–913.1) μg/ml in normal controls, *P* = 0.033; **Figures 1A, B**]. In addition, higher CFH levels were also observed in individuals with the TT or CT genotypes than in those with the CC genotype [TT+CT versus CC: 278.4 ± 68.7 μg/ml versus 236.9 ± 58.7 μg/ml in patients with IgAN, *P* = 0.025; 330.4 (251.2–366.3) μg/ml versus 243.1 (183.6–278.6) μg/ml in normal controls, *P* = 0.002; **Figures 1C, D**]. However, there was no significant difference in the circulating C3a levels among individuals with different genotypes of rs12614 (**Figures 1E, F**). The results indicated that the AP activation was milder in patients with IgAN and normal controls who carried the rs12614-T allele.

### Prediction of damaging effect of rs12614

Three different algorithms were applied to calculate the probability that rs12614 would induce alterations in CFB protein function (**Supplementary Table 6**). As a missense variant in exon 2 of *CFB*, the minor allele of rs12614 (T) caused a change in the amino acid sequence, where residue 32 changed from arginine (R) to tryptophan (W). SNP rs12614 was annotated as a regulatory variant with score 2b and a score of 0.882 after RegulomeDB analysis (**Supplementary Table 6**). Moreover, this variant was predicted to be damaging, with a score of 0.985 by PolyPhen-2 and a score of 0.02 by SIFT analysis. All of these results suggested that this variant was likely to influence the expression and function of CFB protein.

### Effects of rs12614 on the stability of CFB protein and its binding affinity to C3b

**Figure 2:**
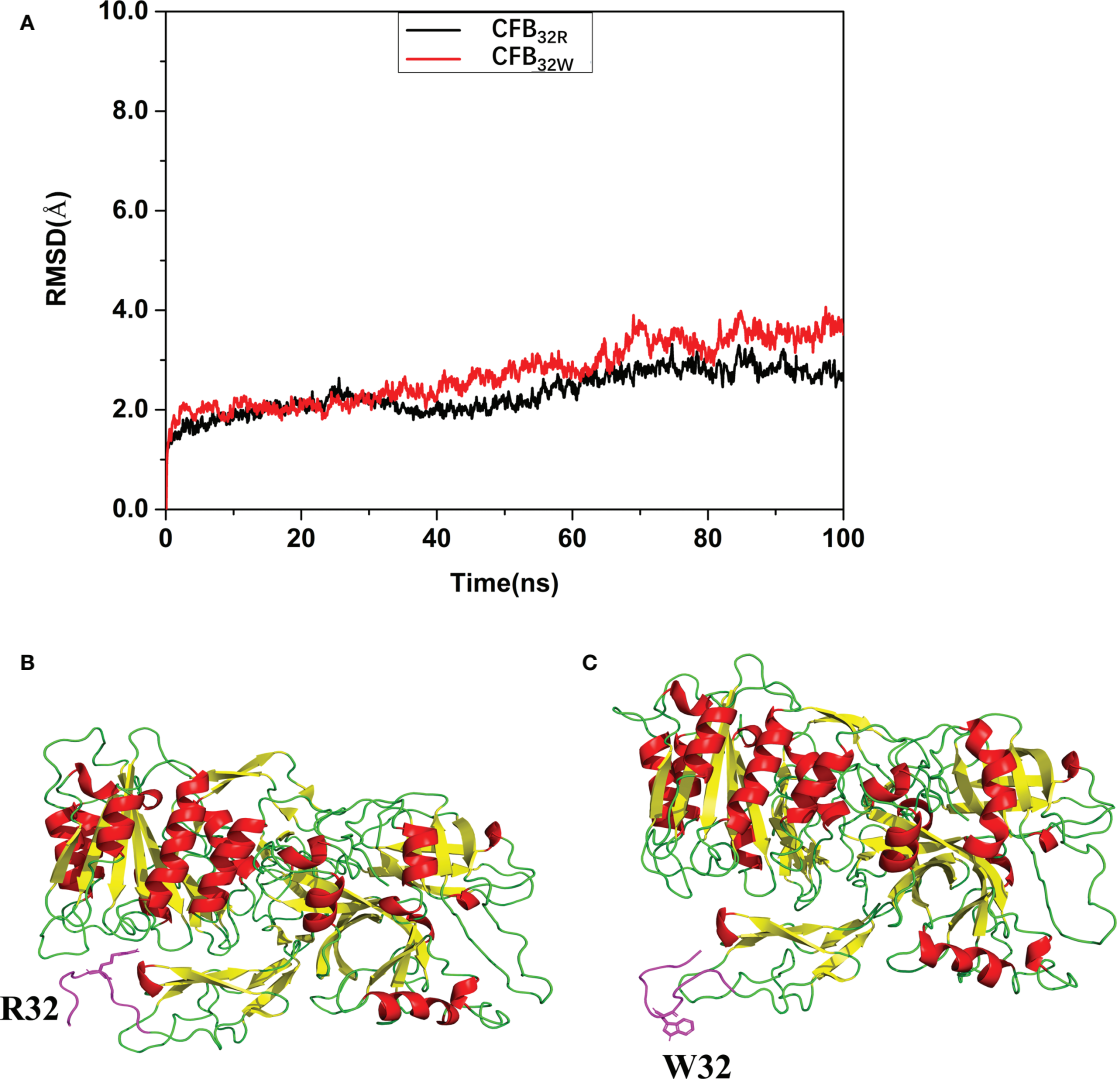
Effect of rs12614 (R32W mutation) on the stability of the CFB protein. **(A)** System flexibility analysis of CFB_32R_ and CFB_32W_. A molecular docking search was performed followed by all-atom, explicit water MD simulations. The root mean square deviation (RMSD) of the backbone of CFB_32W_ (3.5107 Å) was larger than that of CFB_32R_ (2.7145 Å). **(B, C)** Final stable structures of CFB_32R_ **(B)** and CFB_32W_ **(C)**. A clustering strategy was applied to the MD trajectory. The cluster center after the equilibrium of the system was selected as the final stable complex of luteolin with CFB_32R_ and CFB_32W_. The 10-residue-length loops containing the R32 and W32 are colored in purple.

To calculate the effect of rs12614 (R32W) on the protein structure and stability of CFB protein, molecular dynamic simulation and molecular docking were performed. As shown in **Figure 2A**, the root mean square deviation of the backbone of CFB_32W_ (3.5107 Å) was larger than that of CFB_32R_ (2.7145 Å). A clustering strategy was applied to the molecular docking trajectory. The cluster center after the equilibrium of the system was selected as the final stable complex of luteolin with CFB_32R_ and CFB_32W_ (**Figures 2B, C**). In CFB_32R_, R32 maintained the hydrogen bond interaction with amino acids in the upper loop region. However, in CFB_32W_, the hydrogen bond interaction disappeared, and the loop area where W32 is located was far from the upper loop area and swings freely. These differences probably exert a certain negative effect on the stability of CFB_32W_. The results suggested that the R32W mutation could cause a conformational change and reduce the structural stability of the CFB protein.

**Figure 3:**
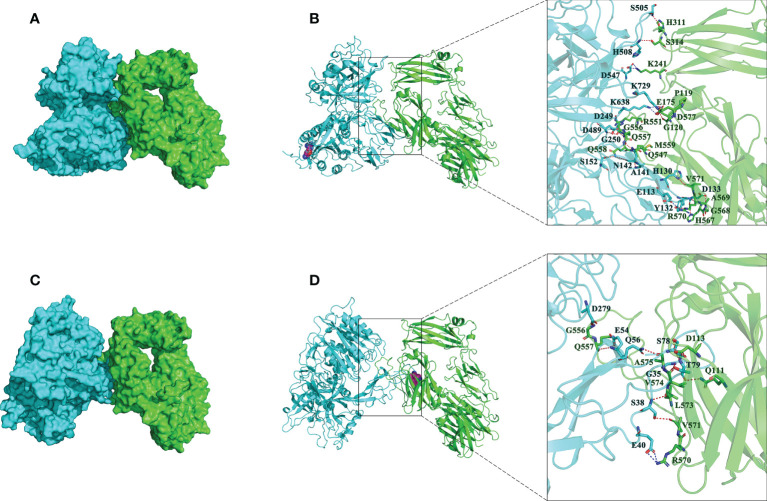
Interactions between best poses of CFB with amino acids of C3b. **(A)** The surface binding model of CFB_32R_ with C3b. **(B)** The detailed interaction between CFB_32R_ and C3b. **(C)** The surface binding model of CFB_32W_ with C3b. **(D)** The detailed interaction between CFB_32W_ and C3b. CFB is colored with cyan, and C3b is colored green. The residues in CFB are colored in cyan. The residues in C3b are colored in green. Residue R32W in CFB is shown as a magenta sphere. The red dashes represent hydrogen bond interactions, and the blue dashes represent salt bridges.

Then, the impact of R32W mutation on binding affinity of *CFB* to C3b was further studied. The binding mode of CFB_32R_ or CFB_32W_ to C3b was shown in **Figure 3**. The binding score of CFB_32W_ and C3b protein (−916.8) was higher than that of CFB_32R_ (−1184.6). The number of amino acids of CFB_32W_ involved in binding to C3b was also markedly lower than that of CFB_32R_. These results indicated that the binding affinity of CFB_32W_ to C3b protein was weaker than that of CFB_32R_.

### Effect of rs12614 variant on the expression of CFB protein

**Figure 4:**
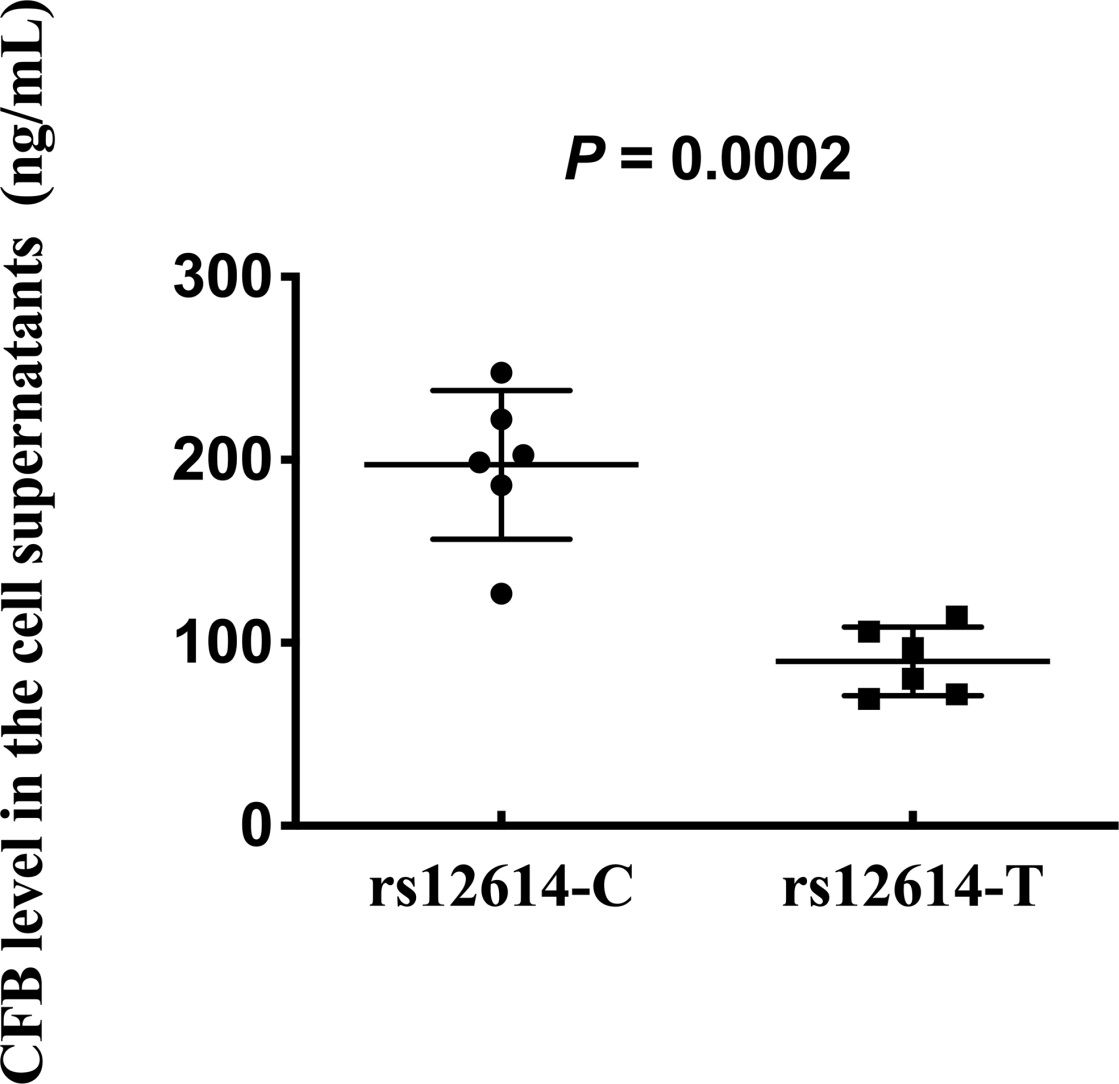
Effect of rs12614 on the expression of CFB protein. Plasmids carrying rs12614-C (CFB_32R_) or rs12614-T (CFB_32W_) were transfected into HEK293 cells. The expression levels of CFB in the cell supernatants were measured by ELISA. The data were replicated in three independent experiments. The bars represent median (interquartile range). The *P*-value was tested by the non-parametric Mann-Whitney *U*-test.

To further investigate whether rs12614 could affect the expression levels of CFB protein, plasmids carrying the rs12614-C (CFB_32R_) or rs12614-T (CFB_32W_) alleles were transfected into HEK293 cells. Compared with CFB_32R_ (197.3 ± 40.7 ng/ml), the expression levels of CFB_32W_ (89.9 ± 18.7 ng/ml) in cell culture supernatants were significantly decreased (**Figure 4**).

### Functional behavior for rs12614 on the formation of C3 convertase

**Figure 5:**
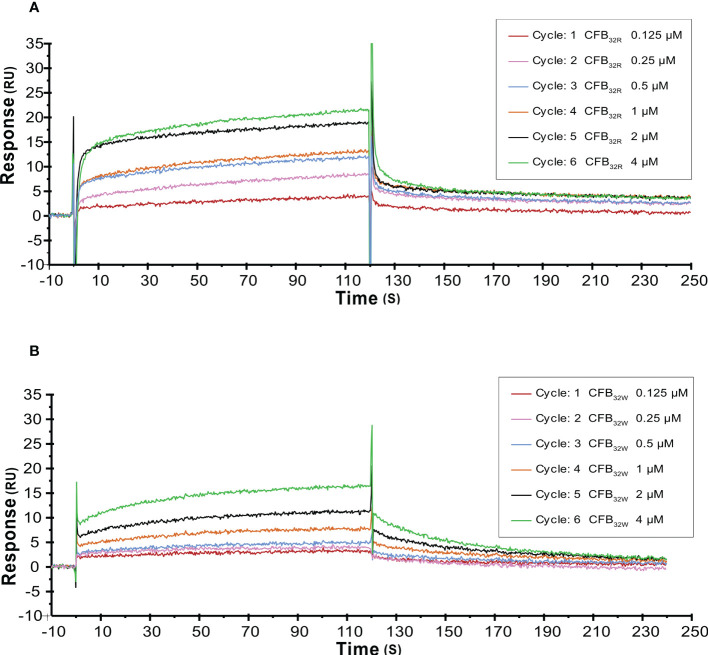
SPR analysis of C3 convertase proenzyme (C3B) formation by CFB_32R_ **(A)** or CFB_32W_ **(B)** C3 convertase proenzyme C3bB is made by the binding of C3 to CFB. Kinetic analysis of the interaction between CFB_32R_ **(A)** or CFB_32w_ **(B)** and C3b was performed by SPR technology with Biacore T1000. One representative set of fitted curves is shown using solution concentrations of 0.125 to 4 µM.

The effect of rs12614 (R32W) on the formation of the proenzyme C3bB was assessed by SPR. C3b was immobilized on a CM5 chip surface. Various concentrations of WT or mutant CFB were flowed over immobilized C3b in the presence of Mg^2+^ and without CFD. Specific binding results showed that CFB_32W_ presented a lower resonance unit value than CFB_32R_ (**Figures 5A, B**). In addition, CFB_32W_ had a higher K_D_ value (CFB_32W_, 2.25 μM; CFB_32R_, 0.61 μM) (**Supplementary Table 7**). The results indicated that the binding affinity of CFB_32W_ to C3b was lower than that of CFB_32R_.

**Figure 6:**
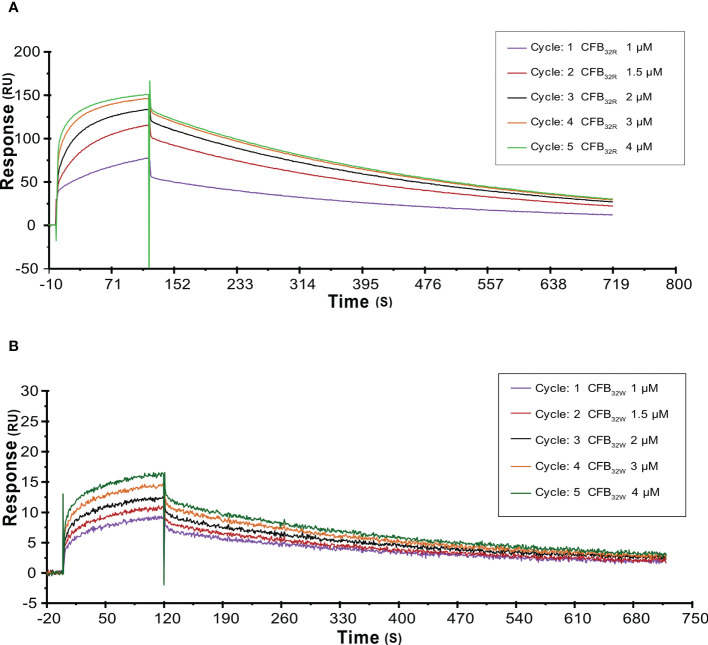
SPR analysis of activated C3 convertase (C3Bb) formation by CFB_32R_ **(A)** or CFB_32W_ **(B)** C3 convertase proenzyme (C3bB) could be activated by complement factor D yielding the C3 convertase (C3bBb). Kinetic analysis of the interaction between CFB_32R_ **(A)** or CFB_32W_ **(B)**, complement factor D, and C3b was performed by SPR technology with Biacore T1000 equipment. One representative set of fitted curves is shown using CFB_32R_ or CFB_32W_ solution concentrations of 1.0, 1.5, 2.0, 3.0, and 4.0 μM.

To further analyze the effects of rs12614 (R32W) on the formation of activated C3 convertase, CFB_32R_ or CFB_32W_ was flowed over the surface of the C3b-immobilized chip, and CFD was added. CFB_32W_ showed a four-fold higher K_D_ value than CFB_32R_ (CFB_32W_, 318.0 nM; CFB_32R_, 77.3 nM) (**Supplementary Table 7**). Compared with CFB_32R_, CFB_32W_ bound to less C3b, leading to decreased convertase formation (**Figures 6A, B**). There was an obvious difference of enzyme formation between CFB_32R_ and CFB_32W_, whereas the decay rates of these two enzymes were similar (**Supplementary Table 7**). The results suggested that rs12614-T in *CFB* showed an obviously lower binding affinity of CFB to C3b.

## Discussion

Complement activation, especially the AP, plays a critical role in the pathogenesis of IgAN (7, 8). Our previous study showed that rs4151657 significantly increased the risk of IgAN, suggesting that *CFB* was a susceptibility gene for IgAN (13). In this study, we investigated the associations of other five tag SNPs in *CFB* with IgAN and found that rs12614-T (R32W) conferred low risk of IgAN and led to less complement AP activation in IgAN. The rs12614-T not only decreased the expression level of CFB protein but also contributed to its lower binding affinity to C3b, which was probably due to the conformational change and reduced stability of the CFB protein. This work has demonstrated the potential vital role of the *CFB* polymorphism in IgAN and verified the functional implication of rs12614 in the complement AP activation.

The complement system has recently been recognized as a bridge between innate and adaptive immunity, and it can control the clearance of pathogens, cellular debris, and immune complexes (15). Previous studies reported that inherited or acquired genetic mutations of complement AP components contributed to the pathogenesis of several glomerular diseases, such as IgAN, lupus nephritis, C3 glomerulonephritis, and aHUS (25). Because of the important role of complement activation in the pathogenesis of IgAN, some patients with IgAN have been treated with new investigational medicinal products named LNP023 that targeted the AP or lectin pathway, to stop or slow the course of disease (26). Although the clinical trials are ongoing and results are pending, current reported data supported the pathogenic role of complement activation in IgAN (27). Notably, LNP023, as the first orally available small molecule, could inhibit the function of the CFB protein and interfere with the AP activation (28). This agent is in a phase III clinical trials (NCT04578834) and is expected to be a promising targeted drug for IgAN treatment (16). The CFB protein is an initial molecule that promotes AP activation, which is encoded by the *CFB* gene located in the MHC III region of chromosome 6 (6). Numerous genetic studies have demonstrated that rs12614 within *CFB* confers susceptibility to several immune and infectious diseases, such as age-related macular degeneration (29) and chronic hepatitis B (30, 31). Our previous study has reported that *CFB* gene was a novel susceptibility gene for IgAN and might be involved in the development and progression of IgAN by affecting the activation of complement pathway (13). In this study, we found another SNP rs12614 and a protective haplotype within *CFB* conferred the susceptibility of IgAN, demonstrating the importance of *CFB* variants in the development of IgAN.

The genotype–phenotype analysis in this study showed that patients with IgAN with the rs12614-T allele presented with high serum C3 levels and less intense mesangial C3 deposition, which were indicators of mild complement activation. Similarly, several studies found that decreased serum C3 levels and intense mesangial C3 deposition could predict the poor prognosis of IgAN (32, 33), suggesting that the complement activation may be an essential player in the pathogenesis of IgAN. However, no association was observed between rs12614 and the renal outcome of IgAN, and a longer follow-up time may be needed for further elucidation.

To assess the effect of rs12614 on the complement AP activation, we measured the serum levels of key AP components, including CFB, CFH, and C3a. The results showed that the protective allele T of rs12614 was correlated with decreased serum CFB levels and increased CFH levels. During the complement AP activation, CFH is a negative regulatory molecule and competes with CFB to inhibit the formation of C3 convertase, and C3a is a cleavage product of complement activation (34). Recently, Zhou et al. reported that the *CFB* expression was enhanced in both glomeruli and tubulointerstitium in patients with IgAN compared with the healthy controls (14). In addition, some studies suggested that the plasma levels of complement proteins, such as CFB, CFH, Ba, and C4, were increased in IgAN, demonstrating the pathogenic role of the complement system for IgAN (9, 10). Interestingly, a high CFH level in the urinary was also observed in patients with IgAN and was closely correlated with severe renal CFH deposition, suggesting that CFH could be a useful indicator of kidney injury in IgAN (35, 36). Considering the correlation of the rs12614-T allele with the decreased CFB levels, increased CFH and C3 levels, and less intense mesangial C3 deposition, the protective allele T of rs12614 may contribute to the attenuating the complement AP activation in IgAN.

To further confirm the role of rs12614-T (R32W) in IgAN, we conducted a functional experiment. Our results showed that the R32W mutation in *CFB* not only decreased the protein expression level of CFB but also attenuated its binding affinity to C3b and then reduced the formation of C3 convertase. These effects were probably due to the conformational change and decreased stability of the CFB protein. Therefore, the non-synonymous variant rs12614 could affect complement AP activation in patients with IgAN *via* its effect on CFB function. Similar to our findings, another mutation in *CFB*, namely, R32Q (rs641153), was found to be protective against AMD due to its lower C3b affinity and lower efficiency at amplifying complement AP (22).

In summary, we found that the rs12614 variant located in *CFB* was significantly associated with a lower risk of IgAN and mild complement AP activation. The rs12614-T allele played a protective role against IgAN, probably by decreasing the protein expression level of CFB and formation of C3 convertase. Our findings can provide genetic and experimental evidence to support the involvement of R32W in attenuating complement AP activation in IgAN and may shed new light on the personalized treatment for IgAN.

## Data availability statement

The original contributions presented in the study are included in the article/**Supplementary Material**. Further inquiries can be directed to the corresponding author.

## Ethics statement

This study was reviewed and approved by the Ethics Committee of The First Affiliated Hospital, Sun Yat-sen University. The patients/participants provided their written informed consent to participate in this study.

## Author contributions

ML, D-CS, and S-ZF conceived the study, generated the original hypothesis, and designed the experiments. D-CS, S-ZF, ZZ, LC, MW, and D-YF collected the clinical data of patients, performed experiments, and analyzed the data. D-CS and S-ZF wrote the article. ML and X-QY provided critical revision of the article. All authors contributed to the article and approved the submitted version.

## Funding

This work was supported by the National Key Research and Development Project of China (No. 2016YFC0906100), the Guangdong-Hong Kong Joint Laboratory on Immunological and Genetic Kidney Diseases (No. 2019B121205005), National Natural Science Foundation of China (Nos. 81920108008, 81770661, and 82170714), the GuangDong Basic and Applied Basic Research Foundation (No. 2021A1515111054), and the Young and Middle-aged Talents Program of The First Affiliated Hospital, Sun Yat-sen University.

## Acknowledgments

We are thankful to all the study participants. We thank Professor Jianjun Liu for reviewing the article and providing constructive suggestions.

## Conflict of interest

The authors declare that the research was conducted in the absence of any commercial or financial relationships that could be construed as a potential conflict of interest.

## Publisher’s note

All claims expressed in this article are solely those of the authors and do not necessarily represent those of their affiliated organizations, or those of the publisher, the editors and the reviewers. Any product that may be evaluated in this article, or claim that may be made by its manufacturer, is not guaranteed or endorsed by the publisher.
