# Corrigendum: Functional variant rs12614 in *CFB* confers a low risk of IgA nephropathy by attenuating complement alternative pathway activation in Han Chinese

**DOI:** 10.3389/fimmu.2022.1098003

**Published:** 2022-11-24

**Authors:** Dian-Chun Shi, Shao-Zhen Feng, Zhong Zhong, Lu Cai, Meng Wang, Dong-Ying Fu, Xue-Qing Yu, Ming Li

**Affiliations:** ^1^ Department of Nephrology, The First Affiliated Hospital of Sun Yat-sen University, Guangzhou, China; ^2^ NHC Key Laboratory of Clinical Nephrology (Sun Yat-sen University) and Guangdong Provincial Key Laboratory of Nephrology, Guangzhou, China; ^3^ Division of Nephrology, Guangdong Provincial People’s Hospital, Guangdong Academy of Medical Sciences, Guangzhou, China

**Keywords:** IgA nephropathy, complement factor B, case-control study, alternative pathway, polymorphism

In the published article, there was an error regarding the affiliations for all authors. As well as having affiliation 1, they should also have “^2^NHC Key Laboratory of Clinical Nephrology (Sun Yat-sen University) and Guangdong Provincial Key Laboratory of Nephrology, Guangzhou, China”.

The author Ming Li should also have “^3^Division of Nephrology, Guangdong Provincial People’s Hospital, Guangdong Academy of Medical Sciences, Guangzhou, China”.

The authors apologize for this error and state that this does not change the scientific conclusions of the article in any way. The original article has been updated.

## Publisher’s note

All claims expressed in this article are solely those of the authors and do not necessarily represent those of their affiliated organizations, or those of the publisher, the editors and the reviewers. Any product that may be evaluated in this article, or claim that may be made by its manufacturer, is not guaranteed or endorsed by the publisher.

