# Supplementary material for: Functional variant rs12614 in CFB confers a low risk of IgA nephropathy by attenuating complement alternative pathway activation in Han Chinese
Source: Front Immunol. 2022 Oct 13;13:973169. doi: 10.3389/fimmu.2022.973169 (PMC9606215; doi:10.3389/fimmu.2022.973169)

Supplementary Table 1. The characteristics of the selected six SNPs

SNP	Allele[a]	Position[b]	Location
rs12614	T/C	31914179	exon 2
rs1048709	A/G	31914935	exon 3
rs537160	T/C	31916400	intron 7
rs541862	G/A	31916951	intron 8
rs4151657	C/T	31917540	intron 10
rs2072633	C/T	31919578	intron 17

[a] Minor/major allele
[b] Derived from the Ensemble (GRCh37.p13) database
SNP, single-nucleotide polymorphism

Supplementary Table 2. Primers for *CFB* variants genotyping

SNP	Primer_Allele FAM	Primer_Allele HEX	Primer_Common
rs1048709	ATCGCTGTCTGCCCACTCCAT	CGCTGTCTGCCCACTCCAC	GCCAATCGCACCTGCCAAGTGAA
rs12614	CCACTCCATGGTCTTTGGCCC	ACCACTCCATGGTCTTTGGCCT	TACCCCCTCCAGAGAGCAGGAT
rs2072633	AAGCATGGCTGTTCCTGCTTGC	GAAGCATGGCTGTTCCTGCTTGT	CCTAGTCTGATTCCTTTAGGTCAGCTA
rs537160	CCAACGATCCTGCTGTTCAACC	CCCAACGATCCTGCTGTTCAACT	CAGGGAGGACCACTTTGTAGTCAAA
rs541862	CAACTTGCTCACCCTGCCAT	CAACTTGCTCACCCTGCCAC	GAAGATAAACTGGCTAAAGGCAGGGAT

SNP, single-nucleotide polymorphism

Supplementary Table 3. Baseline characteristics of study subjects

Characteristics	Cases[d] (N=1333)	Controls (N=1413)
Male (n, %)	670 (50.26%)	696 (49.26%)
Age, year, mean ± SD	34.86 ± 11.20	35.39 ±10.96
Hypertension (n, %)	471 (35.33%)	
Serum creatinine (μmol/l)	108.00 (73.00, 200.75)	
eGFR[a] (ml/min/1.73m^2)	65.23 (30.31, 96.30)	
Serum IgA level[b] (g/l) (n, %)		
> 3.45 g/l	367 (28.58%)	
⩽ 3.45 g/l	917 (71.42%)	
Serum C3 level[c] (g/l) (n, %)		
< 0.79 g/l	287 (22.32%)	
⩾ 0.79 g/l	999 (77.68%)	
Proteinuria (g/day)	1.36 (0.56, 2.93)	
Hyperuricemia	730 (55.90%)	
Hyperlipemia	968 (74.81%)	
Mesangial C3 deposition		
0	274 (20.59%)	
±~1+	392 (29.45%)	
2+	559 (42.00%)	
3+~4+	106 (7.96%)	
Oxford classification		
Mesangial hypercellularity (M1) (n, %)	735 (55.14%)	
Endocapillary hypercellularity (E1) (n, %)	235 (17.63%)	
Segmental glomerulosclerosis (S1) (n, %)	725 (54.39%)	
Tubular atrophy/Interstitial fibrosis (n, %)		
T0	780 (58.51%)	
T1	344 (25.81%)	
T2	209 (15.68%)	

Values are calculated as mean ± SD, median (interquartile range) or frequency (%).
[a]eGFR was calculated by the MDRD formula
[b]3.45 g/l is the higher limit of serum IgA level according to our hospital reference range
[c]0.79 g/l is the lower limit of serum C3 level according to our hospital reference range
[d]A few cases are lack of specific data and we only analyzed those who had the information
SD, standard deviation; eGFR, estimated glomerular filtration rate

Supplementary Table 4. Haplotype analysis of *CFB* gene polymorphisms with the susceptibility of IgAN

Haplotype[a]	Frequency		OR (95 % CI)	P^b	Permutation P^c
	Cases	Controls			
CACC	0.33	0.31	1.10（0.98-1.24）	0.115	0.431
CATC	0.14	0.17	0.75（0.64-0.87）	**1.33E-04**	**0.002**
CGTT	0.05	0.05	0.99（0.78-1.25）	0.900	1.000
TATT	0.44	0.44	1.00	0.996	1.000
CATT	0.04	0.03	1.32（0.99-1.76）	0.059	0.250

Bold characters indicated reaching significance for associations ($P < 0.05$)
[a]Haplotypes consisted of SNPs rs537160, rs541862, rs4151657 and rs2072633
[b]The *P* values were calculated by using logistic regression analysis adjusted for age and gender.
[c]One thousand-fold permutation test.
IgAN, IgA nephropathy; OR, odds ratio; CI, confidence interval

Supplementary Table 5. Cox regression analysis of rs12614 for renal progression

SNP (A/B)[a]	Genotype	Unadjusted model		Adjusted model[b]	
		HR (95%CI)	*P*	HR (95%CI)	*P*
rs12614 (T/C)	CC	1.00 (reference)		1.00 (reference)	
	TC+TT	0.72 (0.29-1.77)	0.468	1.89 (0.74-4.80)	0.182

[a]A: minor allele/B: major allele

[b]Adjusted model was conducted after adjustment for known prognostic factors including age, gender, proteinuria, hypertension and estimated glomerular filtration rate

SNP, single-nucleotide polymorphism；HR, Hazard ratio, CI, confidence interval

Supplementary Table 6. Prediction for the damaging probability of SNP rs12614

Position[a]	Nucleotide	Amino Acid	Polyphen-2[b]	SIFT[c]	SNP score/rank[d]
6:31914179	c.94C>T	p.R32W	0.985	0.02	0.882/2b

[a]Derived from the Ensemble (GRCh37.p13) database
[b]SNP function was predicted by Polyphen-2 (http://genetics.bwh.harvard.edu/pph2/).
[c]SNP function was predicted by SIFT (http://sift.bii.a-star.edu.sg/)
[d]SNP function was predicted by RegulomeDB (https://regulome.stanford.edu/)

Supplementary Table 7. Effect of rs12614 variant on kinetic parameters of proenzyme and activated C3 convertase formation by SPR analysis

	ka[a] (1/Ms)	kd[b] (1/s)	K_D[c] (M)
Proenzyme			
CFB_{32W}	6695	1.50E-02	2.25E-06
CFB_{32R}	14490	8.80E-03	6.08E-07
Activated C3 convertase			
CFB_{32W}	7418	2.36E-03	3.18E-07
CFB_{32R}	31960	2.47E-03	7.73E-08

[a]ka: association constant

[b]kd: dissociation constant

[c]K_D: equilibrium dissociation constant, reciprocal of affinity

CFB_{32W}: Purified CFB protein containing W32; CFB_{32R}: Purified CFB protein containing R32.

SPR, Surface plasmon resonance

Supplementary Figure 1. The LD plot for SNPs of *CFB* measured by r^2. The block structure was estimated using Haploview 4.2, and one LD block has been identified (black solid line).

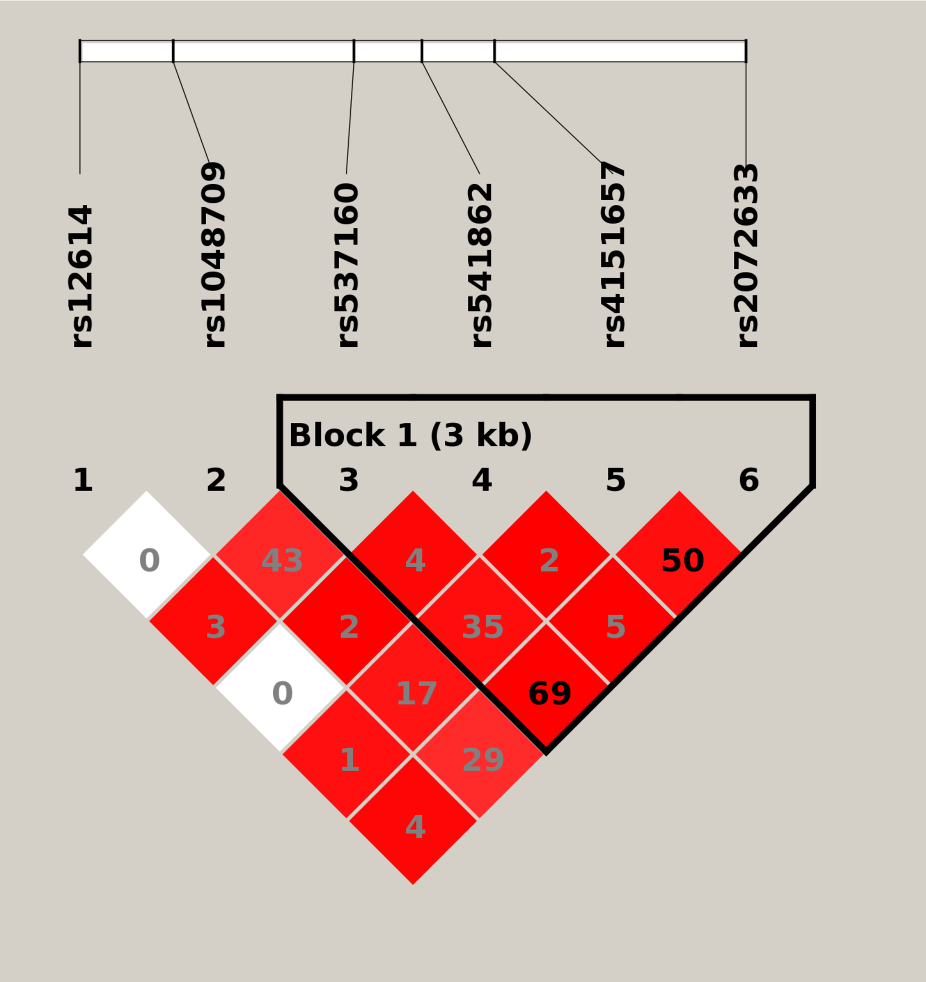

Supplement: Supplementary file 1 [file DataSheet_1.pdf]
